# Antibacterial Mode of Action of the *Daucus carota* Essential Oil Active Compounds against *Campylobacter jejuni* and Efflux-Mediated Drug Resistance in Gram-Negative Bacteria

**DOI:** 10.3390/molecules25225448

**Published:** 2020-11-20

**Authors:** Luc Dedieu, Jean Michel Brunel, Vanina Lorenzi, Alain Muselli, Liliane Berti, Jean Michel Bolla

**Affiliations:** 1 Avignon Univ, INRAE, UMR SQPOV, SporAlim Team, Domaine Saint-Paul–Site Agroparc, 84914 Avignon CEDEX 9, France; luc.dedieu@univ-avignon.fr; 2 Aix-Marseille Univ, INSERM, SSA, IRBA, MCT, UMR_MD1, U-1261, 13005 Marseille CEDEX 5, France; bruneljm@yahoo.fr; 3 Université de Corse, UMR CNRS 6134, SPE Sciences Pour l’Environnement, Campus Grimaldi, 20250 Corte, France; lorenzi_v@univ-corse.fr (L.V.); muselli_a@univ-corse.fr (M.A.); berti_l@univ-corse.fr (B.L.)

**Keywords:** natural compounds, chemical synthesis, efflux pump, ATP monitoring, Elemicin, *E*-methylisoeugenol

## Abstract

Today, an alarming rise of bacterial gastroenteritis in humans resulting from consuming *Campylobacter*-tainted foods is being observed. One of the solutions for mitigating this issue may be the antibacterial activity of essential oils. In the present research, we propose to study the antibacterial activity against *Campylobacter* and other Gram-negative bacteria of *Daucus carota* essential oil and its active molecules. In addition, a few chemically synthesized molecules such as (*E*)-methylisoeugenol, Elemicin, and eugenol were also studied. The results showed that the essential oil itself and its most active component, (*E*)-methylisoeugenol, exhibited bactericidal effects. Similar effects were detected using purified and chemically synthesized molecules. Also, it was observed that the *Daucus carota* essential oil and its active molecules affected intracellular potassium and intracellular ATP contents in *Campylobacter* cells. Inhibition of the membrane bound F_O_F_1_-ATPase was also observed. Eventually, for the first time, the efflux mechanism of active molecules of *Daucus carota* essential oil was also identified in gamma proteobacteria and its specific antibacterial activity against *Campylobacter jejuni* was associated with the lack of this efflux mechanism in this species.

## 1. Introduction

*Campylobacter* is one of the leading causes of bacterial gastroenteritis worldwide [1]. The mode of transmission is documented and is known to originate mainly from the ingestion of undercooked poultry products. *Campylobacter*, mostly *Campylobacter jejuni*, and to a lesser degree, *Campylobacter coli* are isolated in 100% of the layer and broiler flocks. These bacteria colonize the animal’s intestine and are later transferred to the carcass during their processing [2]. Therefore, in order to control the contagion in carcasses, it is important to reduce contamination among animals during their breeding or slaughter. In addition, with ever-increasing multi-drug resistant bacteria, consumers have now been opting for organic food, including antibiotic-free meat and poultry products. Thus, there is an urgent need to develop new strategies to manage contamination. Recently, pertaining to this context, essential oils (EOs) extracted naturally from plants have been highlighted as a promising solution [3]. Many vegetable extracts from herbs and plants from all over the world have been used ancestrally in traditional medicines for different properties, including their antimicrobial activity. Several EOs have antimicrobial activity against Gram-positive and Gram-negative bacteria, yeasts, and filamentous fungi [4,5]. The interest for the antibacterial activity of EOs increased due to the threat toward the food industry, with zoonotic pathogens such as *Salmonella*, *Shigella*, *Listeria*, and *Campylobacter* [6]. However, once a new plant extract has been established which carries an effective anti-pathogenic activity, it is still necessary to identify the active molecule and/or the combination of active molecules and to further determine their mechanism of action. To date, many antimicrobial molecules including phenolic compounds such as vanillin, thymol, and carvacrol have been found in several EOs [7,8,9,10]. They have been studied as natural antimicrobial agents since the use of antibiotics in animal food farms is restricted [7,8,11,12,13]. There are well-documented works of literature that have reported that compounds like eugenol, cinnamaldehyde, and carvacrol alter the ATP level in the cell and cause cell membrane disruption [11,12,13].

In a previous study, a panel of 28 EOs from Corsica was screened for antibacterial activity against five bacterial strains from the species *Escherichia coli*, *Enterobacter aerogenes*, *Pseudomonas aeruginosa*, *Staphylococcus aureus*, and *Campylobacter jejuni*. Results showed that the *Daucus carota* essential oil (DCEO) was one of the more active oils against *C. jejuni* [14]. Moreover, DCEO is specifically active on *C. jejuni* and does not affect the other bacteria screened in this study [14]. The chemical composition of DCEO has been investigated at different stages of development [15] and in different geographical locations, such as Corsica and Portugal [15,16]. In a second study conducted by Rossi et al., DCEO was fractionated by liquid chromatography and the resulting fractions were analyzed by GC and GC/MS. The oxygenated fraction containing the antibacterial activity was further submitted to column chromatography. Among several fractions, two separate fractions containing 97% (*E*)-methylisoeugenol (EMIE) and 98% Elemicin were obtained, respectively. Minimum inhibitory concentration (MIC) of these fractions against *C. jejuni* was 0.125 and 0.250 µg/mL, respectively. Structural analogues have been used to classify the molecular characteristics that are essential for antibacterial activity [9]. To date, however, no information on the type of impact (bactericidal or bacteriostatic), the target or the mechanism of action is available.

In the present study, we examined the mode of action of EMIE and Elemicin and compared it to the mode of action of the entire DCEO. Using *E. coli* efflux pump mutants, we have demonstrated that similar effects are observed with said strains and *C. jejuni* wild type strains suggesting that the target is shared by both species. Decisively, we can state that due to lack of *E. coli*-like efflux mechanism DCEO and its most active component, EMIE, exhibited activity specific to *C. jejuni*.

## 2. Results

### 2.1. Synthesis of Elemicin (**4**) and EMIE (**6**)

**Scheme 1:**
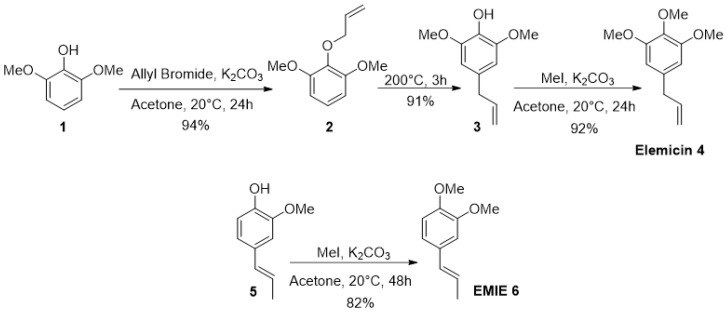
Synthetic pathway for the preparation of Elemicin (**4**) and EMIE (**6**)**.**

As shown in Scheme 1, 2,6-dimethoxyphenol (**1**) was easily protected with allyl bromide to give 2-(allyloxy)-1,3-dimethoxybenzene (**2**, yield, 94%), which underwent a Claisen rearrangement to form 3-(4-hydroxy-3,5-dimethoxyphenyl)-1-propene (**3**). A subsequent methylation of the hydroxy group gave 3-(3,4,5-tri-methoxyphenyl)-1-propene (**4**, Elemicin) in 92% yield. In a similar way, isoeugenol (**5**) was easily methylated in 82% yield to form of the expected compound **6** (EMIE). The corresponding NMR spectra are shown in Supplementary Figure S3.

### 2.2. MIC and MBC Determination

To characterize the antibacterial effect of the DCEO against the *C. jejuni* F38011 strain, the EO was prepared in our laboratory as previously described [14] and the DCEO was fractioned by two column chromatography steps on silica gel as previously described [9]. The MIC of the entire oil, the oxygenated fraction (Oxy-Fr), and the EMIE and Elemicin fractions were determined using the standard broth-dilution method and the agar-dilution method. The MIC in broth and agar indicated as MIC^(L)^ and MIC^(S)^, respectively are given in Table 1. Only the MIC^(L)^ value obtained for the DCEO (500 µg/mL) differs from the MIC^(S)^ (250 µg/mL). In addition, only one dilution difference was observed between the two methods suggesting a good correlation within them.

DMSO alone and the hydrocarbon fraction of EO were found to be inactive, irrespective of the method used. The MBC was also determined using the liquid method. The MBC/MIC ratios were calculated for the active compounds and compared to erythromycin and gentamicin, classically termed as bacteriostatic and bactericidal antibiotics, respectively [17,18]. Carvacrol was used as a non-antibiotic positive control to achieve bactericidal effect [8,12,13,19]. We obtained a ratio of 2 for the DCEO and the Oxy-Fr and a ratio of 1 for the purified EMIE and Elemicin molecules indicating, a bactericidal effect for the entire EO and all the active fractions purified along the purification process (Table 1).

The EMIE and Elemicin molecules were chemically synthesized and their MIC^(L)^ and MBC were tested against the *C. jejuni* F38011 strain. Similar results (125 µg/mL for EMIE and 250 µg/mL for Elemicin) were obtained using the purified and the synthesized molecules (Table 1). Both the methods were used to study commercially available solutions of eugenol and isoeugenol as they are very similar to the active molecules. As observed, the MIC^(L)^ and MIC^(S)^ of Isoeugenol was found to be 250 µg/mL and 125 µg/mL, respectively with only is different by just one dilution. Taken together, these results indicate that all the active compounds had a bactericidal effect and that the purified EMIE molecule and the synthesized EMIE molecule are the most active compounds compared to Elemicin and the Oxy-Fr.

### 2.3. MIC and MBC Determination

To evaluate the effect of *Campylobacter* inoculum size on the MIC^(L)^ of DCEO, Oxy-Fr, and purified molecules, variable inoculum sizes ranging from 1 × 10^4^ to 1 × 10^7^ cells/mL were assessed. Experiments were carried out according to the previously described protocol. As observed, no significant changes were observed in MIC^(L)^ of any test samples (Table 2). MIC^(L)^ of DCEO, the Oxy-Fr fraction and Elemicin with inoculum size of 1 × 10^4^ CFU/mL shifted slightly by one dilution however, MIC findings were the same for EMIE, for all the inoculum size measured.

### 2.4. Characterization of the Rapid Bactericidal Effect

As we observed a bactericidal effect, time-kill assays were performed on mid-log phased *Campylobacter* cells to determine the kinetics of the cell death. DCEO concentrations of 500, 1000, and 2000 µg/mL corresponding to MIC, 2 × MIC, and 4 × MIC respectively, were used to treat the cells for 30 min.

**Figure 1:**
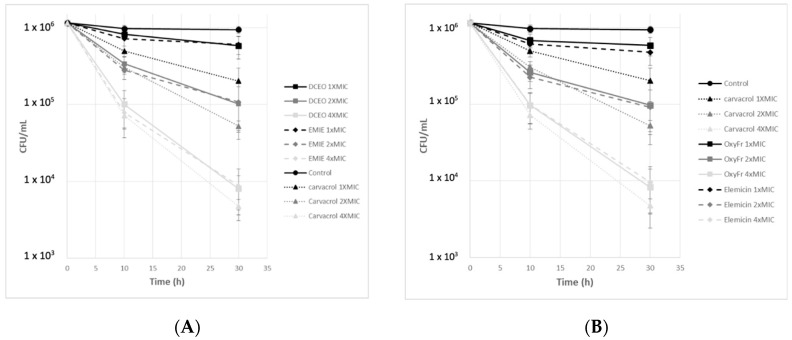
Rapid bactericidal effect of the DCEO and its active molecules against mid-log grown cells of *C. jejuni* F38011 using time kill assays for 30 min with concentrations corresponding to MIC, 2 × MIC, and 4 × MIC. Control cells were treated with DMSO and carvacrol was used as a positive standard. (**A**) effect of the entire DCEO and EMIE. (**B**) effect of Oxy-Fr and Elemicin. Data are the means ± SEM from three independent experiments.

Survival of the bacteria was determined by colony forming units (CFU) counting. The results of test samples were compared to that of negative control i.e., dimethyl sulfoxide (DMSO) treated cells. We observed a dose-dependent response and a DCEO concentration of 2 µg/mL (4 × MIC) induced a strong decrease in CFU count within 30 min (<1% of the bacterial CFU count). Similar results were observed using EMIE (Figure 1A). Same experiments were performed with Oxy-Fr, Elemicin, and carvacrol. Correspondingly, a strong decrease was reached in 30 min with 4 × MIC for all the treatments (Figure 1B). Also, similar results were obtained when experiments were performed on stationary growth phased *Campylobacter* cells (Figure S1). These results suggest that the active molecules acted not specifically on the synthesis of macromolecules necessary during cell growth and are in agreement with the bactericidal effect observed.

### 2.5. Cell Lysis Determination by O.D. Measurements and SDS-PAGE

The decrease in CFU observed could result from bacterial lysis. In order to assess bacterial lysis, cell density was adjusted to 0.5 OD_600_ unit, and cells were treated with DCEO, EMIE, Elemicin, and carvacrol for 30 min. OD measurements were performed after 3, 10, and 30 min and compared with OD measurements of DMSO treated cells (negative standard). SDS and TCA were used to treat samples and were consider as positive standard molecules for cell lysis. We observed that OD_600_ obtained after 30 min for cells treated with DCEO, EMIE, and Elemicin were not significantly different (all *p*-values were >0.2) from OD_600_ obtained with untreated cells (Table S1). On the contrary, OD_600_ obtained after 30 min for cells treated with carvacrol were 20% lower than OD_600_ obtained with untreated cells. Control cells treated with SDS and TCA showed OD_600_ values 90% lower than OD_600_ obtained with untreated cells (Table S1).

**Figure 2:**
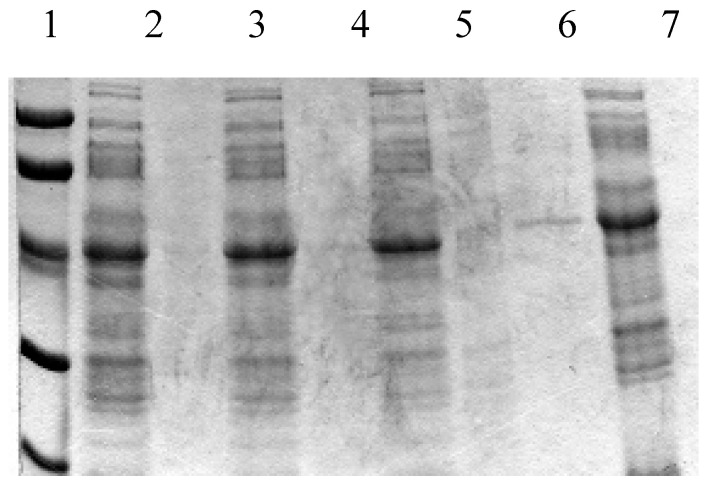
Cell lysis analyzed by SDS-PAGE on cell pellets and supernatants after incubation with different DCEO concentrations. Lane 1, molecular weight; lanes 2, 4, 6, and 8, cell pellets after treatment with DMSO (negative standard), DCEO at 2 × MIC, DCEO at 4 × MIC, and SDS 0.02% (*w*/*v*) respectively. Lanes 3, 5, 7, and 9, supernatants after treatment with DMSO (negative standard), DCEO at 2 × MIC, DCEO at 4 × MIC, and SDS 0.02% (*w/v*) respectively.

To further examine the cell integrity, suspensions containing treated and untreated cells were centrifuged and protein content of the pellets and the supernatants were analyzed by SDS-PAGE. As observed in Figure 2, untreated cells had negligible protein content in the supernatant and most of the protein content was detected in the cell pellet. Same result was obtained with DCEO, EMIE, Elemicin, and carvacrol treated cells. Cell suspensions treated with SDS (positive controls) showed a substantial protein content in the supernatant and almost no protein in the pellet suggesting severe cell lysis. Immunodetection using Omp50 outer membrane protein-specific antibodies [20] was performed on pellets and supernatants of treated and untreated cell suspensions (Figure S2). Although this method is more sensitive, no specific signals were obtained in the supernatant for cell suspensions treated with DCEO, EMIE, and Elemicin indicating that no membrane disruption occurred during the treatment. Collectively, these data showed that there was no cell lysis in these conditions.

### 2.6. Measurement of the Potassium Release

**Figure 3:**
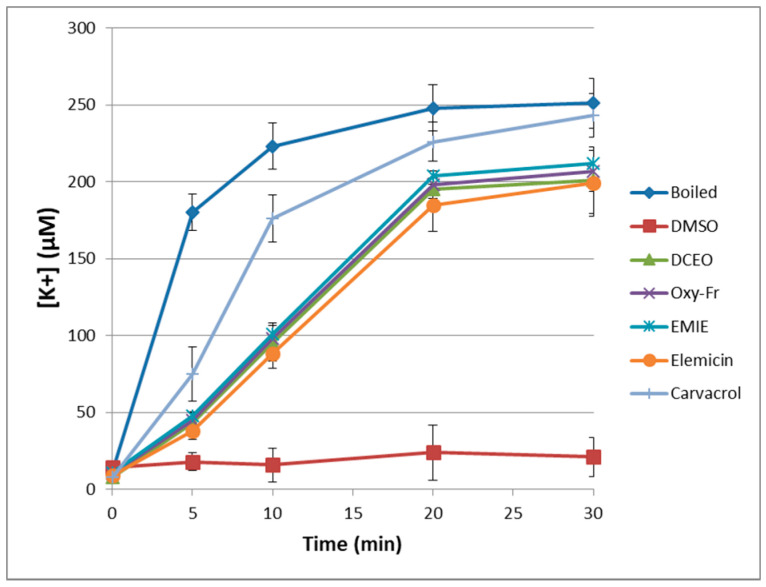
Extracellular potassium level measured by flame emission spectroscopy after treatment with DCEO, oxygenated fraction and purified active molecules. Data are the means ± SEM from three independent experiments.

Although no cell lysis was observed after treatment with DCEO and its active molecules, a partial alteration of membrane functions could explain the cell death. In intact cells, a high intracellular potassium level is maintained compared to the extracellular level. To investigate the cellular potassium gradient integrity, we performed potassium level measurements outside of the cells after treatment (Figure 3). DMSO-treated cells were used as negative standard and heated cells were used as positive standard of cell lysis. We detected a drastic increase of the potassium concentration outside of the cells after treatment with carvacrol. An increase of the potassium concentration was also observed after treatment with DCEO, EMIE and Elemicin. After erythromycin and gentamicin treatment, no increase of the potassium concentration outside of the cells was detected (data not shown). This indicates that in *Campylobacter* the potassium gradient was strongly altered by DCEO and its active molecules.

### 2.7. Cellular ATP Content and ATP Release Measurements

**Figure 4:**
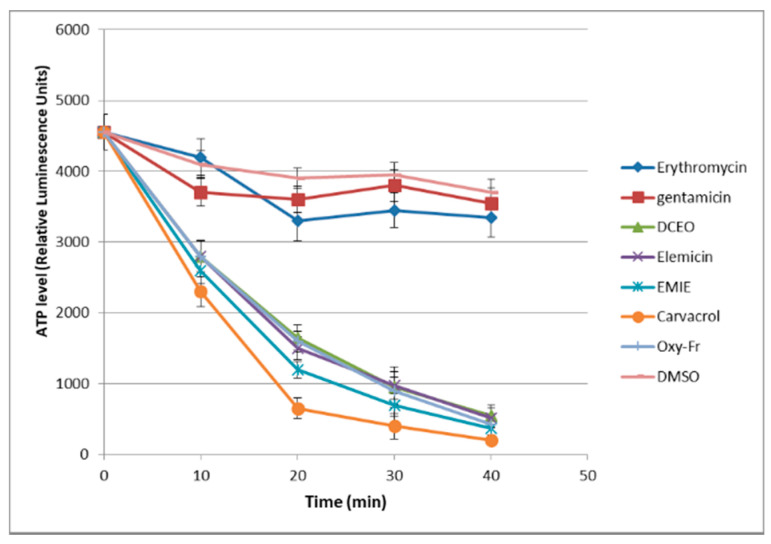
Intracellular ATP level in *Campylobacter* cells after treatment with the entire DCEO, the oxygenated fraction and purified active molecules. Data are the means ± SEM from three independent experiments.

In addition to potassium release, we investigated the ATP concentration inside and outside of cells after treatments. After DCEO, Oxy-Fr, EMIE, Elemicin, and carvacrol treatments, a negligible ATP level was detected outside of cells. The same result was observed for untreated cells (data not shown). On the contrary, ATP concentration inside the cells substantially decreases post-treatment as compared to the DMSO, erythromycin, and gentamicin treated cells (Figure 4).

**Figure 5:**
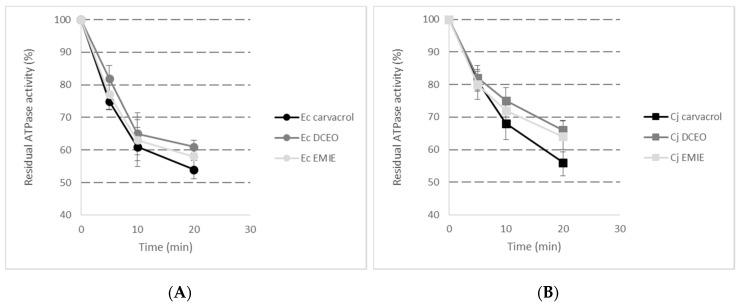
Residual ATPase activity after incubation with DCEO, EMIE, and carvacrol. (**A**) Measured on *E. coli* membrane fractions. (**B**) Measured on *Campylobacter* membrane fractions. Data are the means ± SEM from three independent experiments.

A crucial decrease of the ATP level inside of cells without increase of the extracellular ATP level indicated that ATP molecules were not released from the cells to the medium. Taken together, the measurements of the potassium and ATP levels indicate either a consumption of ATP or an inhibition of the ATP synthesis. To address this question, we investigated the in vitro inhibition of the membrane bound F_O_F_1_-ATPase activity after incubation of purified membrane fractions with the DCEO and its active molecules. *In vitro*, the F_O_F_1_-ATPase can hydrolyze exogenous ATP added in the samples in ADP and inorganic phosphate leading to a decrease in ATP level. Inhibition of this enzyme would result in a slow decrease in ATP concentration [13]. We purified membrane fractions of *C. jejuni* and *E. coli* strains as previously described [21,22]. These preparations were incubated 5, 10 or 20 min with DCEO and ATP was added to the preparation just before ATP levels measurements. Without molecule addition (incubation with DMSO), a rapid and significant decrease in ATP level was observed with membrane fractions of *C. jejuni* and *E. coli* demonstrating the quality of the preparations both containing active F_O_F_1_-ATPase able to maintain its activity (hydrolysis of ATP) for 20 min to perform the tests (Figure 5). Incubation with DCEO and EMIE inhibited the ATPase activity of *E. coli* and *Campylobacter* preparations (Figure 5). After 20 min, the inhibition rate of 4 × MIC carvacrol on *E. coli* ATPase activity was 46%. Similar effect was observed on the *Campylobacter* ATPase with 44% inhibition. After 20 min, the inhibition rate of 4 × MIC EMIE on *E. coli* ATPase activity was 42% and a comparable effect was observed on the *Campylobacter* ATPase with 36% inhibition suggesting an analogous type of effect on the two bacteria.

### 2.8. Specificity of EMIE and Related Molecules Against C. jejuni

Our data showed that the active molecules of DCEO inhibit the action of F_O_F_1_-ATPase in cells of *C. jejuni* and *E. coli*. As previously stated, the specificity of DCEO against *Campylobacter* [14] could not be explained by such a mode of action since F_O_F_1_-ATPase is ubiquitous. Without a definite target in *C. jejuni*, the specificity of DCEO could be explained by a particular resistance mechanism that is absent in *C. jejuni*. To address this query, we determined the MIC^(L)^ of the DCEO and the active molecules against a collection of *C. jejuni* and *E. coli* strains with different efflux levels (Table 3).

According to previous work using the entire DCEO [14], neither DCEO nor the active molecules tested in present study demonstrated any antibacterial activity against *Pseudomonas aeruginosa*, *Enterobacter aerogenes* and *E. coli* wild type strain since the MIC value was higher than 32,000 µg/mL which is over the solubility limit of the compounds (Table 3). When *E. coli* wild type strain was treated with the PABN efflux system inhibitor [23], MIC values of DCEO and Elemicin was found to be 500 µg/mL and 125 µg/mL, respectively.

MIC highly influenced by treatment with PABN indicated that DCEO and its active molecules were transported by a PABN-sensitive efflux mechanism in *E. coli*. The *E. coli* strain AG100A is devoid of AcrAB efflux system. The MIC of DCEO, EMIE and Elemicin in this strain were highly affected by the mutation suggesting that the EO and the molecules have been partly transported by the AcrAB-TolC efflux system. We also used a set of *Campylobacter* strains for MIC determination with or without PABN. MICs for EMIE and Elemicin on *Campylobacter* strain 81,176 which is a well characterized efflux-activated strain [24] were not significantly different from those obtained with the F38011 strain (Table 4). The MIC of DCEO and EMIE was not significantly changed by the use of PABN on this strain or by the use of an isogenic mutant strain deleted from the CmeB efflux component [25]. Compared to untreated cells, only the MIC of Elemicin was significantly changed (by three dilutions) in PABN treated cells. This three dilutions variation is a minor effect compared to the variation obtained with or without PABN for Elemicin MIC in *E. coli* (i.e., at least six dilutions) and compared to the variations obtained with or without PABN for erythromycin MIC (seven dilutions) which is in agreement with the lower activity of Elemicin compared to EMIE. As previously described, erythromycin was used as a positive standard molecule for efflux by a PABN sensitive efflux system and as a negative standard molecule for CmeABC [24]. Interestingly, the MIC of DCEO and EMIE observed against *Helicobacter pylori* was found to be 500 and 250, respectively. Taken together, these results indicate that, in contrast to *E. coli* and other gamma proteobacteria, *Campylobacter* did not resist to DCEO treatment because EMIE, the more active compound, is not extruded by any efflux pump.

## 3. Discussion

Since *Campylobacter* species are detected in 100% of chickens on farms and in more than 75% of commercialized chicken carcasses [2], it is of significant importance to reduce the bacterial load to improve food safety in zoonotic conditions. In this context, it may be useful to study the antimicrobial activity of natural products such as plant-extracted molecules [3]. Here, the MIC of the *Daucus carota* essential oil, its fractions, and chemically synthesized molecules were determined using a solid and a liquid method against *C. jejuni.* The comparison between MIC^(L)^ and MIC^(S)^ yielded very comparable values, suggesting a strong correlation between the two techniques. Similar MIC values were also obtained with purified molecules and chemically synthesized, demonstrating the consistency of the preparations for either chemically synthesized molecules or natural purified molecules. Therefore, we could be assured that no impurities play a major role in the antibacterial activity. In the previous research, MIC of DCEO against *Campylobacter* was studied and EMIE and Elemicin were identified as active molecules [9] but no minimum bactericidal concentration (MBC) value and no mode of action information was obtained because the agar dilution method used to assess the MIC was not suitable for MBC determination. In the present study, it was observed that MBC/MIC ratio of DCEO, EMIE, Elemicin fractions, and chemically synthesized molecules against the *C. jejuni* F38011 strain exhibited bactericidal effect. Moreover, a ratio of 1 (same values for MIC and MBC) was obtained for synthesized and purified EMIE and Elemicin. On the contrary, we obtained ratios of 2 for the entire DCEO and the Oxy-Fr. This resulted probably due to impurities in the entire EO and in the Oxy-Fr. A bactericidal effect has been already observed for other phenolic compounds such as carvacrol and cinnamaldehyde against *Listeria innocua, Bacillus cereus* and *E. coli* O157:H7 [19,26,27].

Using time-kill assays, we observed the same kinetics of death for the DCEO and the EMIE and Elemicin fractions. This supported our hypothesis that these molecules are active compounds. In addition, time-kill experiments performed with DCEO, Oxy-Fr, EMIE, Elemicin, and carvacrol on stationary and mid-log phased *Campylobacter* cells gave similar results signifying that the active molecules did not specifically acted on the synthesis of macromolecules which occur during cell growth viz. peptidoglycan, lipids or protein. This was found to be in agreement with the bactericidal activity observed since differential effects would be observed for bacteriostatic molecules. Since we did not observe cell lysis, studies of essential bacterial functions, such as potassium gradient and ATP production, were carried out to decode the mechanism of action. We found that the potassium gradient and the ATP production processes were both altered. These two phenomena occur with analogous kinetics (linear for 10 min and maximum at 20 min) suggesting that they were not triggered by each other. On the contrary, the kinetics obtained with carvacrol was not similar. Also, the rapid decrease observed in the potassium level could explain ATP depletion observed later. These different kinetics (effect of carvacrol on the potassium release before ATP depletion and effect of DCEO molecules on ATPase and a potassium release at the same time) could indicate different mode of actions. One could hypothesize that potassium release caused by carvacrol induces ATP depletion to restore the potassium gradient which is necessary for bacteria to survive but this hypothesis could not be explained using the results obtained with DCEO molecules.

Depletion of ATP have been already observed with carvacrol on *E. coli* and *L. monocytogenes* [13]. In that study, Gill and Holley showed that incubations of 10 min with 5 mM and 10 mM carvacrol inhibited the ATPase activity of *E. coli* and *L. monocytogenes* by 35% and 40%, respectively, and that a 10 min incubation with 1mM carvacrol had no effect on the ATPase activity. Here, we used a concentration of 4 × MIC corresponding to 1.5 mM carvacrol and observed inhibition of the ATPase of *E. coli* and *Campylobacter* of 39% and 32%, respectively. Using a concentration of 4 × MIC corresponding to 2.8 mM EMIE, we observed inhibition of the ATPase of *E. coli* and *Campylobacter* of 37% and 28%, respectively. However, the rates of inhibition after 20 min of treatment with EMIE were higher, reaching 42% on *E. coli* and 36% on *C. jejuni*.

The species specificity of the DCEO and EMIE antibacterial activity was assessed using gamma proteobacteria including *Enterobacter aerogenes*, *Pseudomonas aeruginosa* and *E. coli*. The results showed that neither the EO nor the purified EMIE molecule tested here showed any antibacterial activity. These results are in agreement with our previous study using the agar dilution method [14]. Moreover, we showed that treatment with the PABN inhibitor on the wild type *E. coli* (strain AG100) and that deletion for the AcrAB efflux system in the *E. coli* (strain AG100A) strongly affected the MIC of DCEO, EMIE, and Elemicin. This suggested that DCEO active molecules are partly transported by the AcrAB-TolC efflux system. Furthermore, for the *E. coli* AG100A strain treated with the PABN efflux system inhibitor, the MIC was similar to that obtained for the PABN treated *E. coli* wild type strain. These results suggested that in *E. coli* the molecules could be transported by at least two efflux systems that are PABN-sensitive. Surprisingly, the increase in susceptibility observed after PABN treatment and after deletion differed for DCEO, EMIE and Elemicin. This could be explained by molecules in the mixture that could interact with the efflux system or compete with EMIE and Elemicin. The lack of antibacterial activity against *E. coli* was also observed in a study conducted by Degirmenci and collaborators using *Daucus carota* juice on the pathogenic O157:H7 *E. coli* strain [28].

On the contrary, in the *Campylobacter* strains, the mutation of the CmeB efflux component and the use of PABN did not significantly modify the MIC values for DCEO and EMIE. Only the MIC of Elemicin was slightly modified (three dilutions) after PABN treatment. This three dilutions variation is negligible compared to the variation obtained with or without PABN for Elemicin MIC in *E. coli* (at least six dilutions) and compared to the variations obtained with or without PABN for erythromycin MIC (seven dilutions) which is not a substrate for CmeABC but is transported by a PABN sensitive efflux system [24]. Cumulatively, these results indicated that, contrary to *E. coli*, *Campylobacter* did not resist DCEO treatment because EMIE, its major and most active compound, is not transported by the efflux pumps present in *Campylobacter*. The MIC observed against *Helicobacter pylori* (500 for the DCEO and 250 for the EMIE) suggested that bacteria phylogenetically close to *Campylobacter jejuni* could be sensitive to DCEO and its active molecules. This was in agreement with the data of a previous study on antibacterial activity of mastic gum essential oil against drug-resistant strains of *Helicobacter pylori* [29]. In addition, as reported by Costa and Iraola [30], new methods of isolation and identification allowed the description of emerging *Campylobacter* species known to be underestimated, that could become other target for the compounds described here.

In this study, a strong decrease of CFU from *Campylobacter* was observed using DCEO, EMIE, and Elemicin at 4 × MIC for 30 min which is a short time. In addition, no effect of inoculum size was observed. These two data could be of importance because they can minimize resistance acquisition during treatment for improving the safety of poultry meat. The use of plant-derived GRAS (generally recognized as safe) antimicrobial molecules as feed supplements for reducing fecal pollutants in chickens have been already tested on *Salmonella enteritidis* and *C. jejuni* [31] and could be considered with DCEO or DCEO molecules. In this context, studies about cytotoxicity, showed that DCEO was not cytotoxic against dendritic cells [16]. Other studies have indicated that Elemicin is not directly toxic but could be metabolized in vivo and produce alkaloid metabolites that could be responsible for hallucinogenic effects [32]. In addition, the effect could be different on mixed populations compared to pure culture as already observed for many molecules including EMIE [33]. The DCEO and EMIE should probably not be considered efficient enough to be used alone. Studies using these molecules in combination with other antibacterial molecules as already described using carvacrol and endolysin LysSA97 against *Staphylococcus aureus* should be considered [34]. Hence, synergistic properties of these compounds with well-established antibiotics should be tested in near future.

In conclusion, the data obtained here highlight the potential of essential oils (EOs) extracted naturally from plants in the context of food safety. In addition, the use of chemically synthesized molecules allowed us to be assured that these molecules are the active compounds and that no impurities play a major role in the antibacterial activity. The bactericidal effect was observed specifically against *Campylobacter* and *Helicobacter* strains, but no effect was observed against gamma-proteobacteria. This provide three interesting outcomes (i) these molecules were not transported efficiently by *Campylobacter* efflux pumps, (ii) the use of antibacterial molecules with specific antibacterial effect should be useful because they exert no effect on human gut microbiota, (iii) the use these molecules could be of interest to target emerging *Campylobacter* species.

## 4. Materials and Methods

### 4.1. General Information

All solvents were purified according to reported procedures, and reagents were used as commercially available. Methanol, ethyl acetate, dichloromethane, and petroleum ether (35–60 °C) were purchased from Merck (Saint Quentin Fallavier, France) and used without further purification. Column chromatography was performed on silica gel (70–230 mesh, Macherey-Nagel, Bethlehem, PA, USA). Commercial solutions of erythromycin, gentamicin, eugenol, isoeugenol, and carvacrol were purchased from Sigma-Aldrich (Saint Quentin Fallavier, France). The EMIE and Elemicin purified fractions and the EMIE and Elemicin chemically synthesized molecules were analyzed by NMR. ^1^H-NMR and ^13^C-NMR spectra were recorded as previously described [9] in CDCl_3_ on an AC 400 spectrometer (Bruker, Billerica, MA, USA) working at 400 MHz and 100 MHz, respectively (the usual abbreviations used in text are: s; singlet, d; doublet, t; triplet, q; quadruplet, m; multiplet). All chemical shifts are given in ppm.

### 4.2. Synthesis of 2-(Allyloxy)-1,3-dimethoxybenzene *(**2**)*

To a solution of 2,6-dimethoxyphenol (syringol, **1**, 3 g, 0.016 mol) in acetone (50 mL) was added potassium carbonate (2.5 g, 0.018 mol) and allyl bromide (2.17 g, 0.018 mol). After stirring the mixture overnight, the solvent was removed and 3N HCl (15 mL) was added to it. The extraction was performed with ethyl acetate, the organic fraction so obtained was washed with brine solution and then dried over anhydrous Na_2_SO_4_. The solvents were removed at reduced pressure. Purification of the residue was performed by flash chromatography (eluent: petroleum ether/ethyl acetate, 15:1, *v*/*v*) to yield compound **2** (4.74 g, 94%); colorless oil: ^1^H-NMR (400 MHz, CDCl_3_): δ (ppm) = 3.84 (s, 6H), 4.47 (d, 2H, *J* = 6.0 Hz), 5.13 (dd, 1H, *J* = 10.4, 1.0 Hz), 5.25 (dd, 1H, *J* = 17.2, 1.6 Hz), 6.03 (m, 1H), 6.49 (d, 2H, *J* = 12.0 Hz), 6.91 (t, 1H, *J* = 12.0 Hz). ^13^C-NMR (100 MHz, CDCl_3_): δ (ppm) = 53.16, 74.65, 105.43, 116.59, 123.36, 135.14, 136.50, 153.81. MS (EI), *m*/*z* 194 (M^+)^.

### 4.3. Synthesis of 3-(4-Hydroxy-3,5-dimethoxyphenyl)-1-propene *(**3**)*

Compound **2** (1.25 g, 6.5 mmol) was heated in the absence of solvents to 200 °C for 3 h and the residue was then later purified using flash chromatography (eluent: petroleum ether/ethyl acetate, 8:1, *v*/*v*) to yield compound **3** (1.2 g, 91%); a colorless oil. ^1^H-NMR (400 MHz, CDCl_3_): δ (ppm) = 2.02 (s, 2H), 5.08 (dd, 1H, *J* = 10.2, 1.0 Hz), 5.10 (dd, 1H, *J* = 17.6, 1.6 Hz), 5.57 (s, 1H), 5.91 (m, 1H), 6.40 (s, 2H). MS (EI), *m*/*z* 194 (M^+^).

### 4.4. Synthesis of 3-(3,4,5-Trimethoxyphenyl)-1-propene (Elemicin) *(**4**)*

To a solution of 3-(4-hydroxy-3,5-dimethoxyphenyl)-1-propene (**3**, 1 g, 5.1 mmol) in acetone (50 mL), potassium carbonate (0.9 g, 6.5 mmol) was added. The suspension was stirred for 10 min and iodomethane (1.1 g, 7.8 mmol) was subsequently added and stirred overnight. The solvent was then evaporated, 3 N HCl (10 mL) was added, and the mixture was later extracted with ethyl acetate. The organic fraction was then washed with water, dried over Na_2_SO_4_ and the solvent was removed. The residue was purified using flash chromatography with petroleum ether/ethyl acetate (10:1, *v*/*v*) as eluent yielding compound **4**; a colorless oil (1.0 g, 92%): ^1^H-NMR (400 MHz, CDCl_3_): 3.30 (d, 2H, *J* = 6.6 Hz), 3.95 (s, 9H), 5.03 (dd, 1H, *J* = 9.6, 1.2 Hz), 5.10 (dd, 1H, *J* = 17.2, 1.6 Hz), 5.91 (m, 1H), 6.34 (s, 2H). ^13^C-NMR (100 MHz, CDCl_3_): δ (ppm) = 40.45, 55.92, 61.30, 104.59, 115.90, 123.58, 135.71, 136.19, 137.13, 153.09. MS (EI), *m*/*z* 208 (M^+^).

### 4.5. Synthesis of (E)-methylisoeugenol (EMIE) *(**6**)*

In a two necked round flask, an amount of 3 g of (*E*)-isoeugenol (**5**, 1.8 mmol) was dissolved in 15 mL of acetone. To this solution, 5 g of K_2_CO_3_ (3.6 mmol) and 5 mL of methyl iodide (3.6 mmol) were subsequently added. The resulting mixture was stirred at room temperature for 48 h. The solvents were then removed in-vacuo, and the crude residue was dissolved in ethyl acetate (50 mL). Later, the residue was washed twice with water (50 mL) and brine (50 mL). The organic layer was dried over MgSO_4_, and the solvent was removed to afford a colorless oil which was purified by flash column chromatography (40 g SiO_2_, eluent petroleum ether: ethyl acetate, 3:1). The product yield was 82%. NMR ^1^H (400 MHz, CDCl_3_): δ ^1^H-NMR (400 MHz, CDCl_3_): 1.86 (d, 3H, *J* = 8.0 Hz), 3.84 (s, 3H), 3.87 (s, 3H), 6.03 (m, 1H), 6.29 (dd, 1H, *J* = 20.0, 1.0 Hz), 6.75-6.89 (m, 3H). ^13^C-NMR (100 MHz, CDCl_3_): δ (ppm) = 55.64, 55.77, 108.45, 110.87, 118.58, 123.58, 130.58, 131.07, 148.10, 148.92. MS (EI), *m*/*z* 179 (M^+^).

### 4.6. Bacterial Strains and Culture Conditions

The bacterial strains used in this study are listed in Table 4. *Campylobacter* strains were routinely grown under microaerophilic conditions on Columbia agar or Mueller-Hinton (MH) agar supplemented with or without 5% sheep blood (Bio-Mérieux, Marcy l’étoile-France). The *Helicobacter pylori* Hp99 strain was routinely grown at 37 °C under microaerophilic conditions on MH agar complemented by 10% sheep blood. Microaerophilic conditions (5% O_2_, 10% CO_2_ and 85% N_2_) were obtained using GENBag microaer (BioMérieux). For experimental requirements (see below) and to improve bacterial growth and survival during all the experiments, liquid cultures of *Cj* and *Hp* were performed in a mixture (50:50) of MH Broth (Oxoid, Dardilly, France) and Minimal Eagle Medium (D-MEM broth, Sigma-Aldrich) [35,36]. *E. coli, Pseudomonas,* and *Enterobacter* strains were routinely grown at 37 °C on Luria Bertani agar (LB agar). *E. coli* AG100A strain is an efflux pump mutant (*acrAB*-) and a kanamycin-resistant derivative of the AG100 strain [37].

**Table 4:** Strains used in this study.

*Genera* and *Species*	Strain Name	Relevant Phenotype	Reference
*Campylobacter jejuni*	F38011	Wild type	[38]
*Campylobacter jejuni*	81176	Wild type, active efflux	[39]
*Campylobacter jejuni*	96C208	Wild type, active efflux	[40]
*Campylobacter jejuni*	81176 CmeB-	CmeB mutant strain	[24]
*Campylobacter jejuni*	96C208 CmeB-	CmeB mutant strain	[24]
*Escherichia coli*	AG100	Wild type	[37]
*Escherichia coli*	AG100A	Km^R^, AcrAB-	[37]
*Helicobacter pylori*	HpJ99	Wild type	[29]
*Enterobacter aerogenes*	ATCC13048	Wild type	[41]
*Pseudomonas aeruginosa*	CIP A22	Wild type	[41]

### 4.7. Fractionation of Essential Oil

DCEO was fractioned by two steps of column chromatography on silica gels as previously described [9]. Briefly, the bulk oil was fractioned by column chromatography on silica gel (ICN 200–500 µm) successively with pentane (*n*-C_5_H_12_) and diethyl ether (Et_2_O) to obtain the apolar hydrocarbon fraction and the oxygenated fraction, respectively. The hydrocarbon fraction was inactive and thus retained to be used as negative controls in further experiments. The oxygenated fraction (Oxy-Fr) was further fractionated using silica gel (ICN 63–200 mm) with an elution gradient *n*-C_5_H_12_/Et_2_O, 95/5 to 0/100. The GC and GC/MS analysis of the 26 sub-fractions obtained by column chromatography permitted the identification of two separate fractions containing EMIE and Elemicin, respectively. As mentioned above [9], The presence of active components was tested by complementary NMR analysis, and GC/MS data verified that these fractions contained more than 95% of antibacterial molecules.

### 4.8. Component Identification

GC-FID and GC-MS analysis of essential oil and its fractions obtained by column chromatography were performed under the experimental conditions described previously [9]. The identification was carried out by comparison of their mass spectra with those compiled in our laboratory-built spectral library, as well as by comparison of their retention indices with those of authentic samples or literature data [42,43]. The GC retention indices (RI) on the polar and non-polar columns were determined relative to the retention time of a series of n-alkanes with linear interpolation. Identification of the major components were ensured by ^13^C-NMR, following a procedure developed and computerized in our laboratories and based on the comparison of ^13^C-NMR chemical shifts of components in the mixture with those of pure compounds compiled in a laboratory-built library [15].

### 4.9. Determination of MIC and MBC

Susceptibility to *Daucus carota* essential oil (DCEO), its purified components, and chemically synthesized molecules is expressed as a minimal inhibitory concentration (MIC) and as minimal bactericidal concentration (MBC). The MIC^(S)^ was performed on agar plates as previously described [14]. For *E. coli*, *Enterobacter*, and *Pseudomonas*, MIC experiments were conducted in 1 mL of liquid media (MIC^(L)^). The liquid media used in said experiment was MH Broth inoculated with 10^6^ cells and incubated at 37 °C for 18 h without agitation. For *C. jejuni*, and *H. pylori*, MIC^(L)^ experiments were conducted in 10 mL volumes of a mixture (50:50) of MH Broth (Oxoid) and Minimal Eagle Medium (D-MEM broth, Sigma-Aldrich) inoculated with 10^7^ cells and incubated at 37 °C for 18 h under agitation in microaerophilic condition. For *Campylobacter*, the size of inocula from 10^4^ to 10^7^ cells were checked. MIC (µg/mL) was defined as the lowest concentration required to completely inhibit the growth of bacteria. Inhibition was observed visually. For all experiments, the DCEO, the DCEO fractions, and the chemically synthesized molecules were used two-fold diluted in dimethyl sulfoxide (DMSO) in order to solubilize in the media. When specified, PABN was used at 10 µg/mL for *Campylobacter* and *Helicobacter*, and at 15 µg/mL for the other bacteria. MBC was defined as the lowest concentration required to kill more than 99% of the bacterial inoculum. The MBC/MIC ratios were calculated for the active compounds including erythromycin and gentamicin, classically termed as bacteriostatic and bactericidal antibiotics, respectively [20,21]. If the ratio MBC/MIC was ≤4, the effect was considered bactericidal and if the ratio MBC/MIC was >4, the effect was described as bacteriostatic [20].

### 4.10. Time-Kill Assays

*Campylobacter* cells were grown in MH Broth and D-MEM (50:50). Cells were harvested at mid-log phase, centrifuged, and resuspended in phosphate buffer saline (PBS), pH 7.4. For time-kill assays, 1 mL containing 5 × 10^6^ cells were incubated with DMSO (negative standard) or with the active compounds at 4 × MIC two-fold diluted in DMSO. CFUs were counted after 10, 30, and 60 min and compared with those obtained with the negative standard.

### 4.11. Effect on Bacterial Lysis

Bacterial lysis was investigated on *Campylobacter* cells grown in MH Broth and D-MEM (50:50). The cells were harvested at mid-log phase and resuspended in PBS, pH 7.4 to achieve a cell density equivalent to 0.5 OD_600_. The cell pellets were then incubated with DMSO (negative standard) or with the DMSO two-fold diluted active compounds. Cells were also treated with Sodium-dodecylsulfate (SDS) and trichloroacetic acid (TCA) as positive standard molecules for cell lysis. Bacterial lysis was assessed by measuring OD_600_ after 10, 30 and 60 min. Values were compared with those obtained with the negative standard. Bacterial lysis was also investigated by SDS-PAGE protein content analysis. Briefly, cells (5 × 10^8^ CFU/mL) were incubated with DMSO or active compounds for 10, 30 and 60 min and then harvested by centrifugation at 10,000× *g* for 10 min. The harvested pellets were frozen at −20 °C prior to analysis. The supernatants were precipitated using trichloroacetic acid (TCA) and later centrifuged to recover the proteins. The extracted proteins were also frozen at −20 °C prior to analysis.

### 4.12. Determination of the Intra- and Extra-Cellular Potassium Content

Mid-log phased *Campylobacter* cells were grown in MH Broth and D-MEM (50:50). Cells were centrifuged and resuspended to obtain a cell density of 1 OD_600nm_. Cells were energized by the addition of 0.2% (*w*/*v*) glucose and incubated with DMSO (negative standard) or two-fold diluted active compounds. After variable time intervals, cells were centrifuged, and respective supernatants were immediately cooled at −80 °C for further analysis. The cell pellets so obtained were treated under agitation with buffer containing 10% (*w*/*v*) TCA and 2 mM EDTA for 2 min. Post-treatment, the disrupted cells were centrifuged, and their resulting supernatants were immediately cooled at −80 °C for further analysis. Intra- and extra-cellular potassium levels were determined by Atomic Absorption Spectrometry using a flame photometer (VARIAN (Agilent), Les Ulis, France) set to read potassium concentrations. Values were extrapolated against a standard calibration curve of KCl.

### 4.13. Determination of the Intra- and Extra-Cellular ATP Content

ATP concentrations were determined in mid-log phased *Campylobacter* cells grown in MH Broth and D-MEM broth (50:50) and membrane extracts of *C. jejuni* and *E. coli* prepared as mentioned previously [21,22]. In both cases, ATP concentrations were determined using the rLuciferin-Luciferase ATP assay kit (Promega, Lyon, France). The reaction was assayed over 10 ms period using a chemiluminometer (Yelen S.A., Marseille, France). For two sets of experiments, three independent assays were performed, and suitable controls were included to ensure that none of the elements interfered with the assays. The intracellular ATP contents were presented as relative luminescence units (RLUs) and compared with the RLU of DMSO treated cells. The Residual activity of the F_O_F_1_-ATPase was deduced from the ratio of the RLU obtained with treatment with tested molecules to the RLU of the negative standard (DMSO).

## Figures and Tables

**Table 1:** Susceptibility and type of antibacterial effect of the DCEO, the DCEO fractions, and synthesized molecules against the *C. jejuni* F38011 strain. Data resulted from three independent experiments.

	MIC^(S)^ *	MIC^(L)^ *	MBC *	MBC/MIC	Effect
DMSO	>1000	>1000	>1000	ND	ND
DCEO	250	500	1000	2	Bactericidal
Oxy-Fr	250	250	500	2	Bactericidal
HC-Fr	>1000	>1000	>1000	ND	ND
EMIE-Fr	125	125	125	1	Bactericidal
Elemicin-Fr	250	250	250	1	Bactericidal
EMIE	ND	125	125	1	Bactericidal
Elemicin	ND	250	250	1	Bactericidal
Erythromycin	0,125	0,125	1	8	Bacteriostatic
Gentamicin	0,5	0,5	1	2	Bactericidal
Carvacrol	ND	31.25	62.5	2	Bactericidal
Eugenol	250	250	250	1	Bactericidal
Isoeugenol	125	250	250	1	Bactericidal

* MIC and MBC values are expressed in µg/mL. MIC^(S)^ values were obtained with the solid method and MIC^(L)^ values were obtained with the liquid method. MBC/MIC ratios were calculated with the MIC^(L)^ values and was used to determine the type of antibacterial effect (see materials and methods section). Oxy-Fr, oxygenated fraction; HC-Fr, hydrocarbon (apolar) fraction; EMIE-Fr, EMIE purified fraction; Elemicin-Fr, Elemicin purified fraction; ND, Not determined.

**Table 2:** Effect of the inoculum size on MIC.

Inoculum Size	1 × 10^4^	1 × 10^5^	1 × 10^6^	1 × 10^7^
DCEO-MIC	250	500	500	500
Oxy-Fr-MIC	125	250	250	250
EMIE-MIC	125	125	125	125
Elemicin-MIC	125	250	250	250

Effect of the inoculum size on the MIC^(L)^ values for the DCEO, the oxygenated fraction (Oxy-Fr), and the synthesized molecules against the *C. jejuni* F38011 strain. MIC values are expressed in µg/mL. Inoculum size ranged from 1 × 10^4^ to 1 × 10^7^ cells per mL. Data resulted from three independent experiments.

**Table 3:** MIC of DCEO and active molecules against Gram negative bacteria.

Strain	PABN	DCEO	EMIE	Elemicin	Erythromycin *
Wild type *E. coli* (AG100)	−	32,000	32,000	32,000	ND
	+	500	125	500	ND
AcrAB^-^ *E. coli* (AG100A)	−	500	1000	1000	ND
	+	500	125	250	ND
Wild type *C. jejuni* (F38011)	−	500	125	250	0.125
Efflux activated *C. jejuni* (81176)	−	500	250	500	0.25
	+	250	125	62.5	0.0015
CmeB^-^ *C. jejuni* (81176B)	−	250	125	250	0.125
	+	125	62.5	62.5	0.0015
*Helicobacter pylori* (HpJ99)	−	500	250	ND	ND
*Enterobacter aerogenes* (ATCC13048)	−	32,000	32,000	ND	ND
*Pseudomonas aeruginosa* (CIP A22)	−	32,000	32,000	ND	ND

* Erythromycin was used as a positive standard molecule for efflux by a PABN sensitive efflux system and as a negative standard molecule for CmeABC activity. Data resulted from three independent experiments.

## Supplementary Materials

The following are available online. Figure S1: Kinetics of bactericidal effect, Figure S2: Outer membrane protein detection, Figure S3: NMR spectra of synthesized compounds, Table S1: Effect of compounds on cell integrity.
