# Supplementary material for: Antibacterial Mode of Action of the Daucus carota Essential Oil Active Compounds against Campylobacter jejuni and Efflux-Mediated Drug Resistance in Gram-Negative Bacteria
Source: Molecules. 2020 Nov 20;25(22):5448. doi: 10.3390/molecules25225448 (PMC7699865; doi:10.3390/molecules25225448)
Supplement: Supplementary file 1 [file molecules-25-05448-s001.pdf]

Supplementary Data

Figure 1. Kinetics of bactericidal effect. Rapid CFU decrease with the DCEO and its active molecules against stationary phase grown cells of *C. jejuni* F38011 using time kill assays for 30 minutes with concentrations corresponding to MIC, 2x MIC, and 4x MIC. Control cells were treated with DMSO and carvacrol was used as a positive standard. A: effect of the entire DCEO and EMIE. B: effect of Oxy-Fr and elemicin. Data are the means +/- SEM from three independent experiments.

A

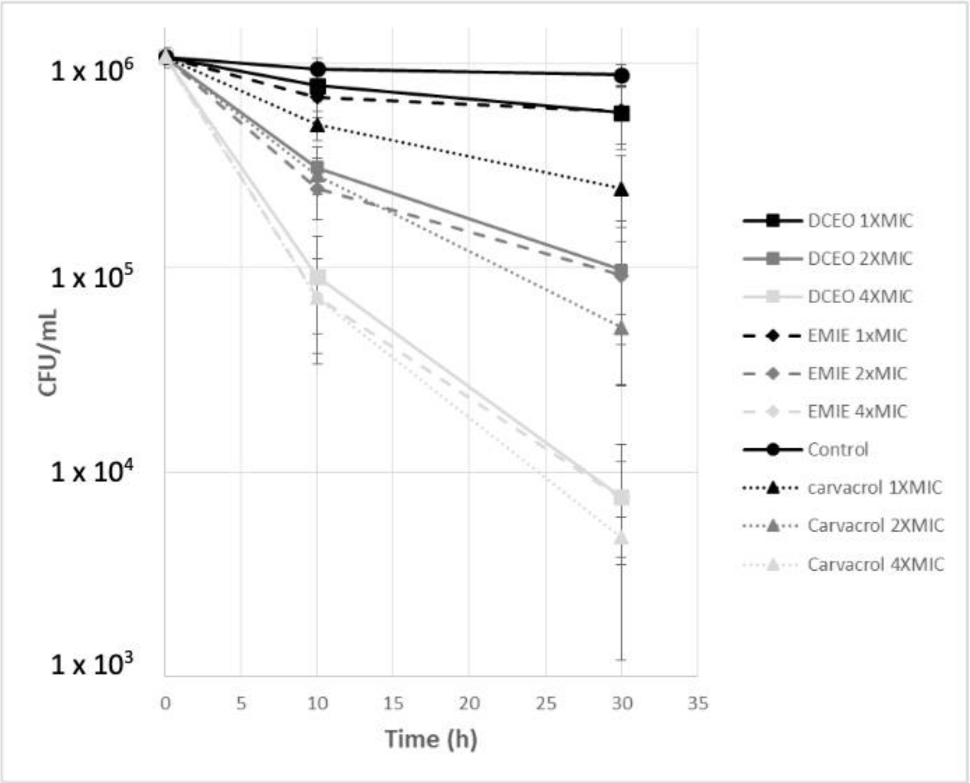

B

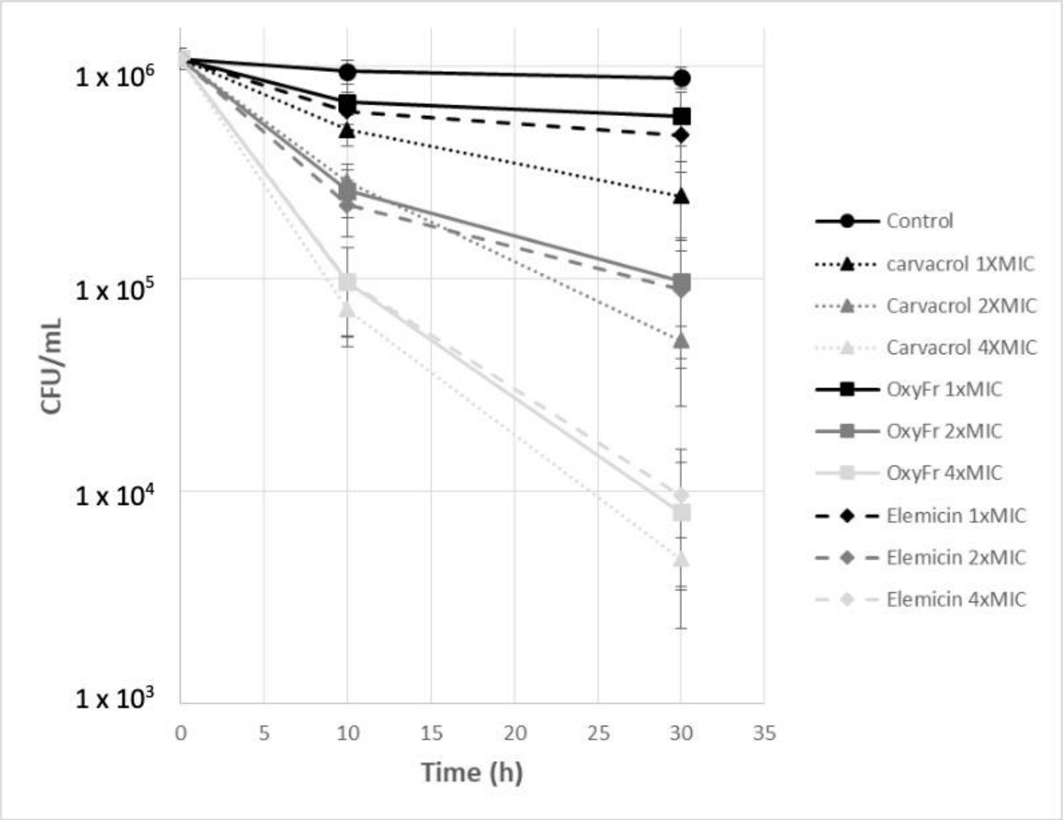

Figure S2: Outer membrane protein detection. Immunodetection using Omp50 outer membrane protein specific antibodies on *Campylobacter* cells treated with 4xMIC DCEO and 4xMIC EMIE. DMSO and SDS were used as negative and positive standards, respectively.

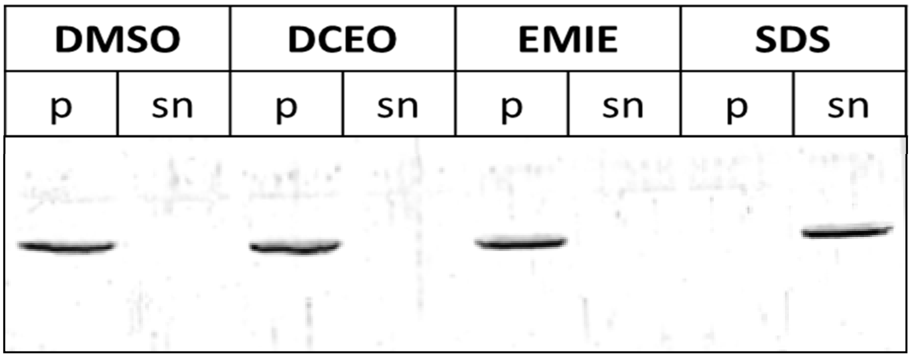

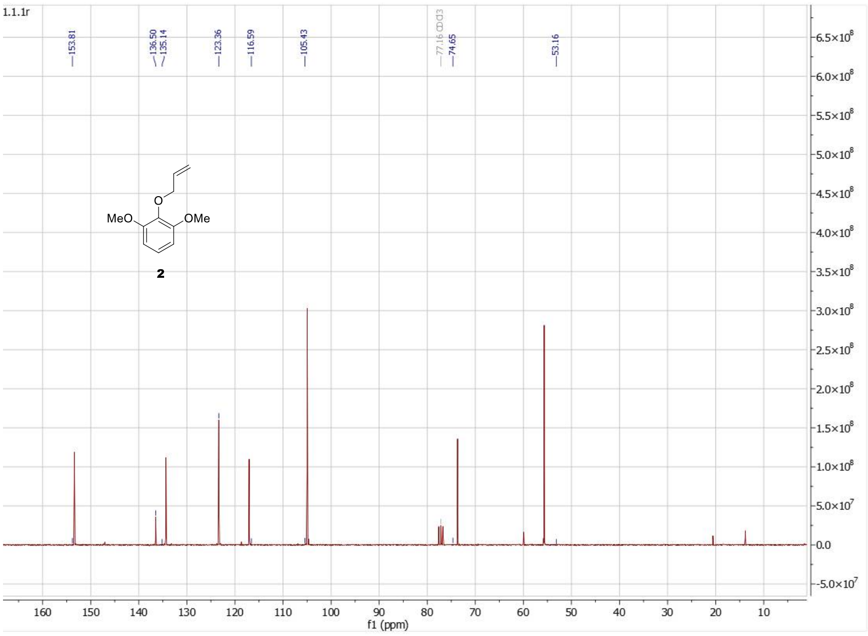
1.1.1r
153.81
136.50
135.14
123.36
116.59
105.43
77.16 CDCl3
74.65
53.16
O
MeO
OMe
2
f1 (ppm)

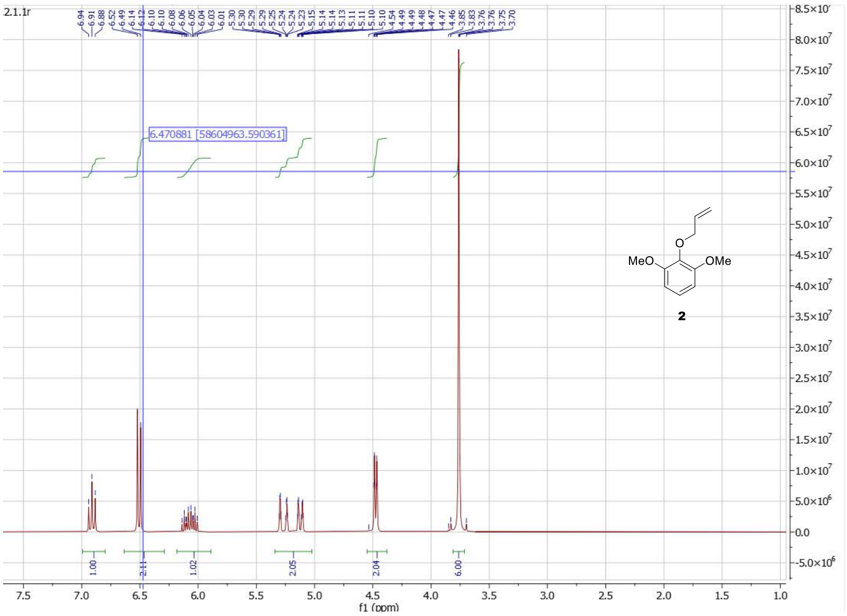
2.1.1r
6.94 6.91 6.88 6.52 6.49 6.14 6.12 6.10 6.10 6.08 6.06 6.05 6.04 6.03 6.01 5.30 5.30 5.29 5.25 5.24 5.24 5.23 5.15 5.14 5.14 5.13 5.11 5.11 5.10 5.10 4.54 4.49 4.49 4.48 4.47 4.47 4.46 3.85 3.83 3.76 3.75 3.70
6.470881 [58604963.590361]
O
MeO
OMe
2
1.00
2.11
1.02
2.05
2.04
6.00
f1 (ppm)

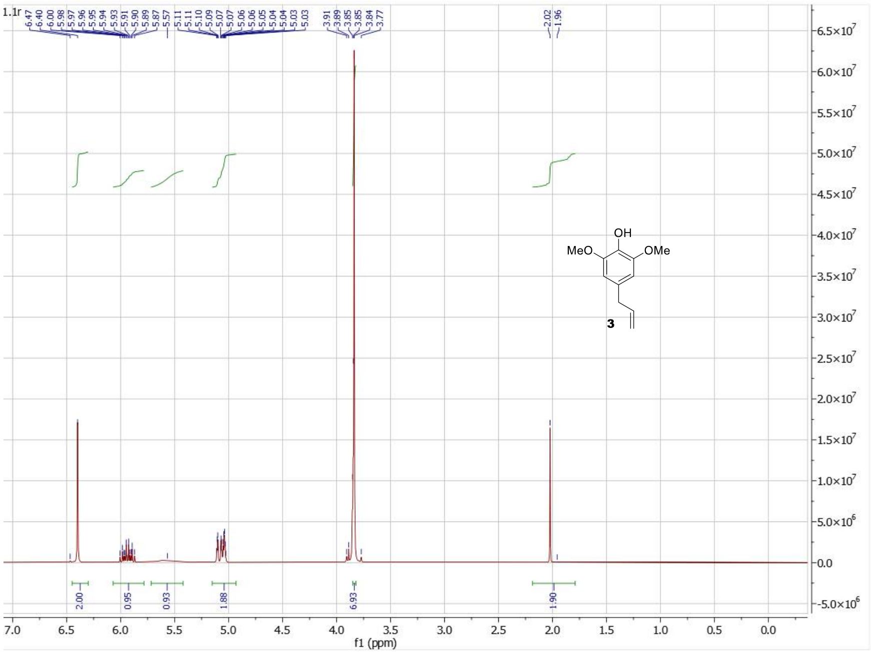

OH
MeO
OMe
3
f1 (ppm)

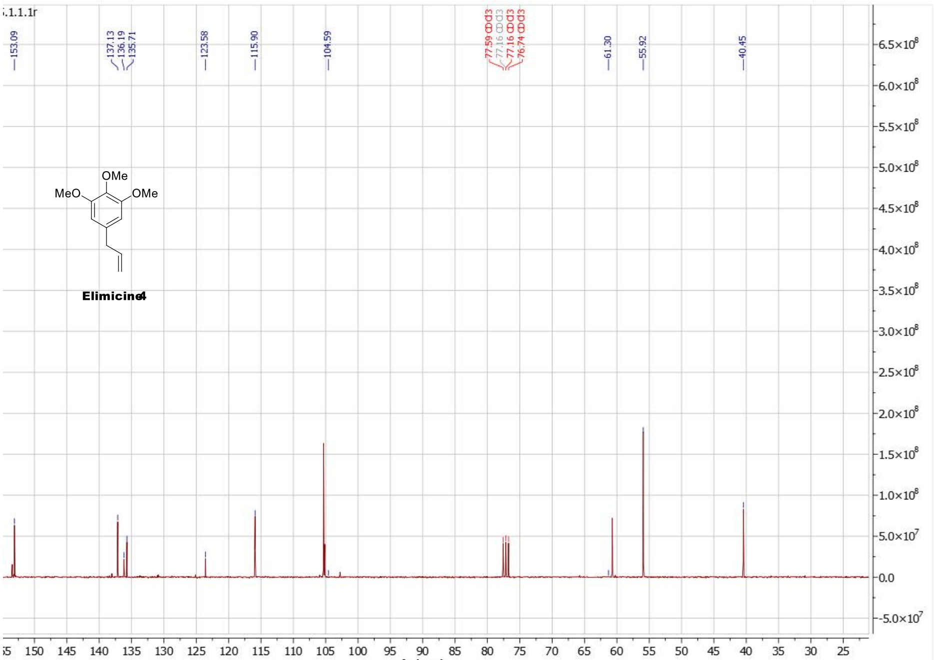

OMe
MeO
OMe
Elimicine4

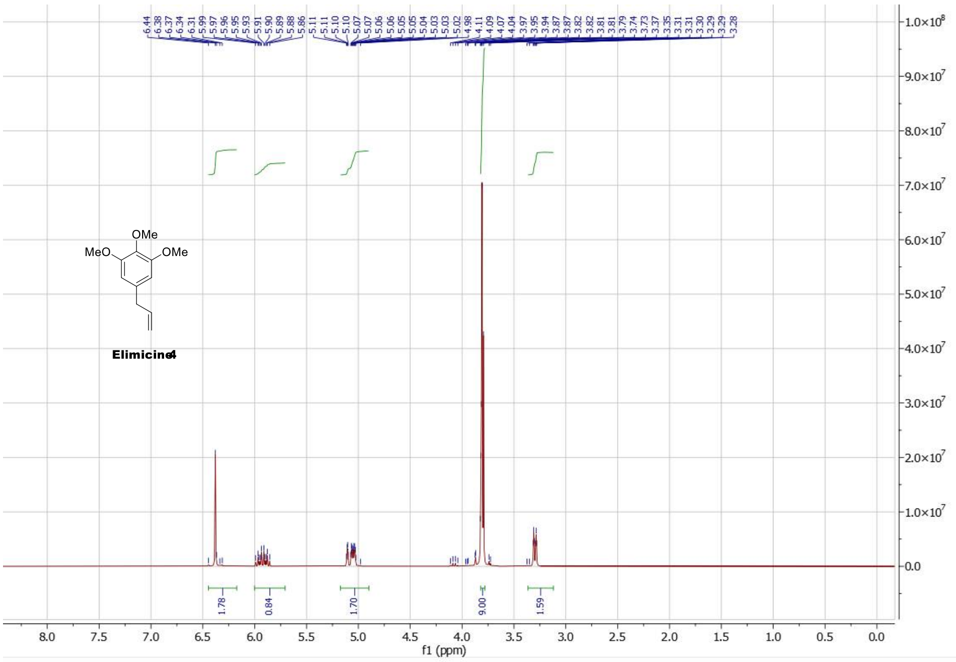
OMe
MeO
OMe
Elimicine4
f1 (ppm)

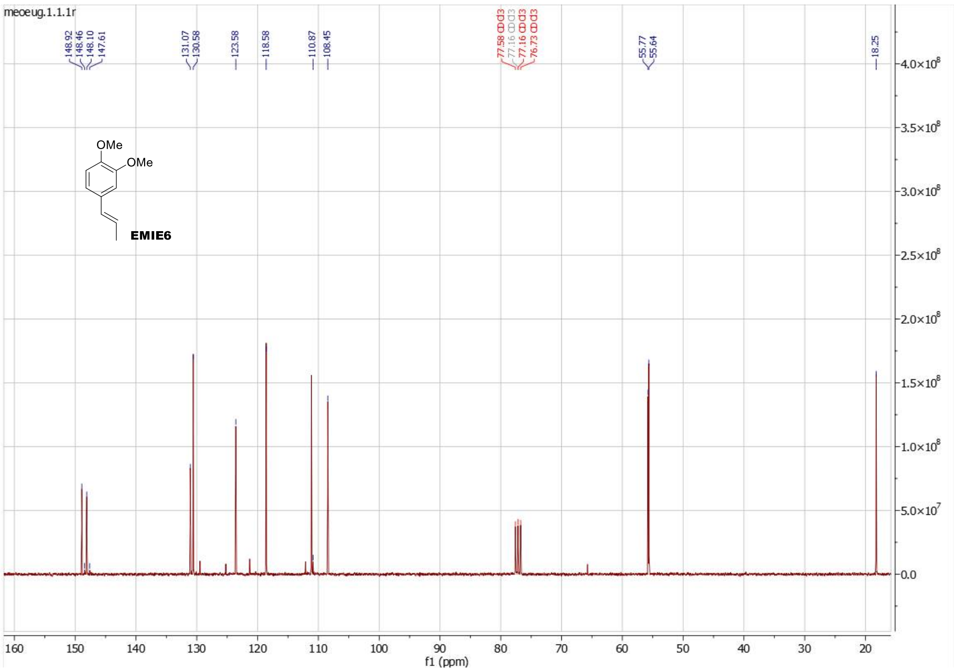
meoeug.1.1.1r
OMe
OMe
EMIE6
f1 (ppm)

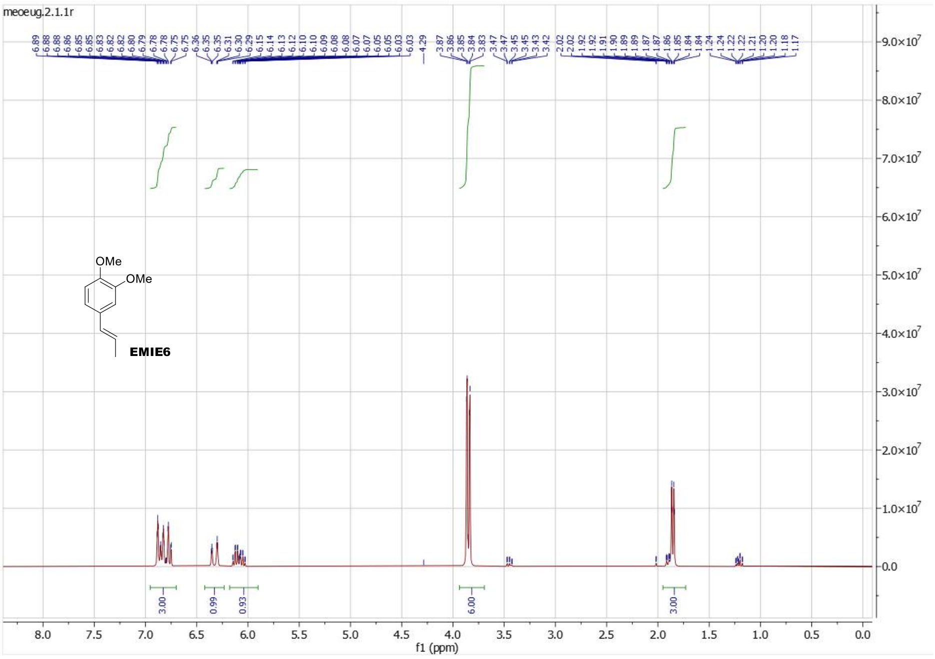
meoeug.2.1.1r
OMe
OMe
EMIE6
f1 (ppm)

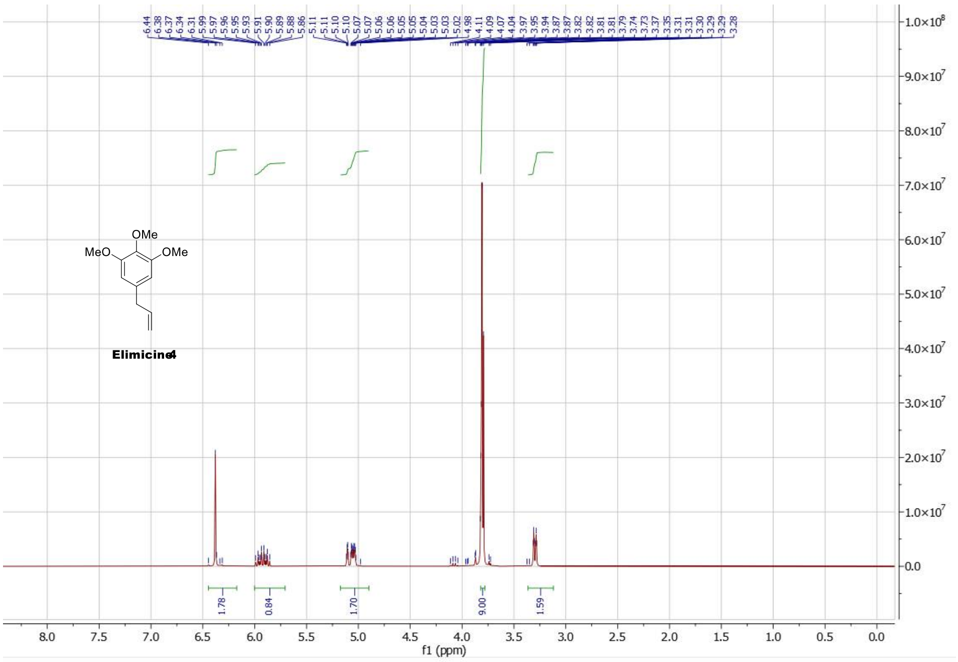

Table 1. Effect of compounds on cell integrity; Optical Density at 600 nm after cell treatment with DCEO, EMIE, elemicin, and carvacrol at 4 x MIC. SDS: Sodium Dodecyl Sulphate; TCA: Tri chloroacetic acid. Data are the means +/- SEM from three independent experiments.

Time (min)	DMSO	DCEO 4MIC	EMIE 4MIC	Elemicin 4MIC	Carvacrol 4MIC	SDS 0,01%	TCA 0,15%
0	0.497±0.012	0.502±0.011	0.501±0.017	0.497±0.014	0.499±0.021	0.498±0.023	0,494±0.017
3	0.504±0.011	0.498±0.012	0.509±0.013	0.498±0.019	0.474±0.012	0.202±0.042	0.078±0.031
10	0.502±0.014	0.501±0.014	0.494±0.016	0.501±0.013	0.438±0.014	0.082±0.028	0.065±0.024
30	0.492±0.018	0.492±0.019	0.491±0.021	0.494±0.024	0.403±0.017	0.062±0.011	0.054±0.015
